# Completing a genomic characterisation of microscopic tumour samples with copy number

**DOI:** 10.1186/s12859-023-05576-7

**Published:** 2023-11-30

**Authors:** Joel Nulsen, Nosheen Hussain, Aws Al-Deka, Jason Yap, Khalil Uddin, Christopher Yau, Ahmed Ashour Ahmed

**Affiliations:** 1 Weatherall Institute for Molecular Medicine, University of Oxford, Oxford, UK; 2 Nuffield Department for Women’s and Reproductive Health, University of Oxford, Oxford, UK; 3 Singula Bio Ltd., Oxford, UK; 4 University of Birmingham, Birmingham, UK; 5 Health Data Research UK, London, UK; 6 Oxford Biomedical Research Centre, National Institute of Health Research, Oxford, UK

**Keywords:** Cancer genomics, Copy number, Microscopic samples

## Abstract

**Background:**

Genomic insights in settings where tumour sample sizes are limited to just hundreds or even tens of cells hold great clinical potential, but also present significant technical challenges. We previously developed the DigiPico sequencing platform to accurately identify somatic mutations from such samples.

**Results:**

Here, we complete this genomic characterisation with copy number. We present a novel protocol, PicoCNV, to call allele-specific somatic copy number alterations from picogram quantities of tumour DNA. We find that PicoCNV provides exactly accurate copy number in 84% of the genome for even the smallest samples, and demonstrate its clinical potential in maintenance therapy.

**Conclusions:**

PicoCNV complements our existing platform, allowing for accurate and comprehensive genomic characterisations of cancers in settings where only microscopic samples are available.

**Supplementary Information:**

The online version contains supplementary material available at 10.1186/s12859-023-05576-7.

## Background

A principal driving force behind each cancer is a repertoire of genomic changes known as somatic alterations [1, 2]. Understanding how these alterations drive cancer is one of the central aims of cancer genomics [3], and efforts in this field have brought about clinical benefits including improved patient stratification, new prognostic biomarkers and an arsenal of new therapies [4, 5]. Most somatic alterations of clinical significance are either small somatic mutations (SSMs) or copy number alterations.

Copy number alterations (CNAs) generally refer to large segments of the genome being either duplicated or deleted. They have been found to drive the cancer phenotype [6, 7], play a prominent role in cancer evolution [8], and hold substantial prognostic value [9, 10]. They are particularly important in genomically unstable cancer types such as ovarian [11], oesophageal [12] and gastric cancers [13]. Due to this importance, researchers have developed many algorithms to call somatic CNAs from sequencing and array data, including ASCAT [14], Sequenza [15], CNVkit [16], ABSOLUTE [17], Control-FREEC [18], OncoSNP-SEQ [19], and TITAN [20], among others. In addition to determining the total number of alleles at each locus in the genome, many of these algorithms also determine the numbers of each the two parental alleles. By convention, these are referred to as the major and minor copy numbers. Such methods are said to call allele-specific CNAs. This added allele-specific information allows researchers to identify more subtle CNAs such as copy number neutral loss of heterozygosity (LOH) [14], which can have important clinical implications in cancer [21].

While the analysis of bulk tumour samples is now routine, recent research has turned towards microscopic settings where samples of interest comprise as few as tens of cancer cells, and bulk samples may be unavailable. These settings, such as minimal residual disease (MRD), tumour initiating cells (TICs) and circulating tumour cells, can hold great clinical potential [22–25]. However, accurately characterising cancer genomes from these microscopic samples is often infeasible with bulk sequencing approaches. Single cell sequencing can provide a solution, especially in cases where intra-sample heterogeneity is of primary interest. Indeed, various algorithms exist to call CNAs from shallow single cell whole genome sequencing (WGS) data, including Ginkgo [26], AneuFinder [27], CHISEL [28] and Alleloscope [29]. However, single cell methods are limited in their ability to reliably identify SSMs [30], which are complementary to CNAs and can be highly consequential for therapeutics [31]. An ideal platform for sequencing microscopic tumour samples would provide both accurate SSMs and CNAs for a complete genomic characterisation. Moreover, many single cell CNA-calling methods rely on aggregating data across thousands of cells, and are therefore unsuitable in settings where total sample size is limited to tens of cells.

To get genomic insights from these microscopic tumour samples, we recently developed a specialised sequencing platform, DigiPico [32]. We have previously described how DigiPico can be used to investigate active sub-clonal mutational processes in cancer in samples as small as thirty cancer cells [32]. Moreover, we estimate that DigiPico has a 76% sensitivity and 95% specificity to detect SSMs (Additional file 1: Table S1). Given the biological and prognostic importance of somatic CNAs, we sought to leverage DigiPico’s unique features to obtain allele-specific somatic CNAs, thus providing a complete genomic characterisation. To that end, we developed a CNA detection pipeline specifically for DigiPico sequencing data, which we call PicoCNV.

### Implementation

**Fig. 1:**
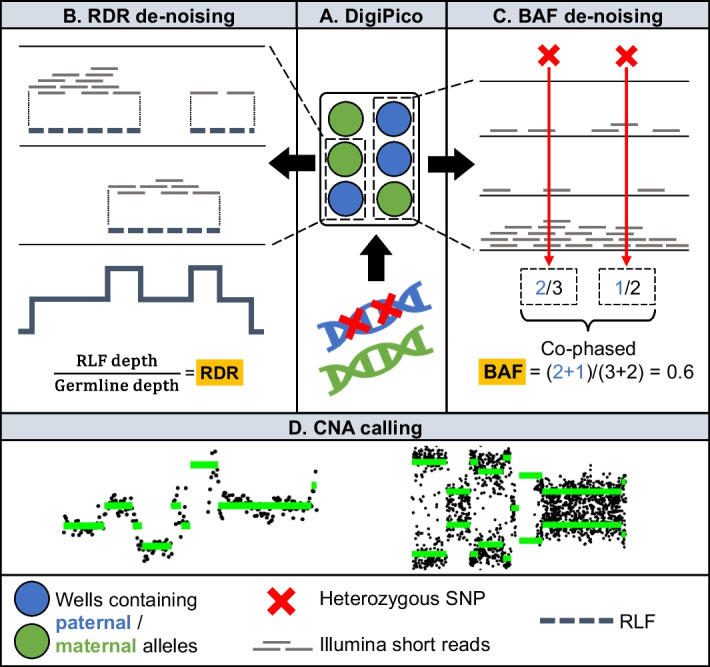
PicoCNV pipeline overview. **A.** Large fragments of tumour DNA are distributed into wells such that each genomic locus is covered by at most one fragment per well, before being sequenced. **B.** Co-local short reads within each well are combined to create reconstructed large fragments (RLFs). Calculating the RDR from the RLF depth counteracts variations in the Illumina short read sequencing depth between fragments. **C.** Nearby SNPs supported by overlapping sets of wells come from the same allele. Calculating the BAF from well counts for haplotype blocks counteracts variations in sequencing depth. **D.** The de-noised RDR and BAF data are used to call allele-specific copy number alterations

**Fig. 2:**
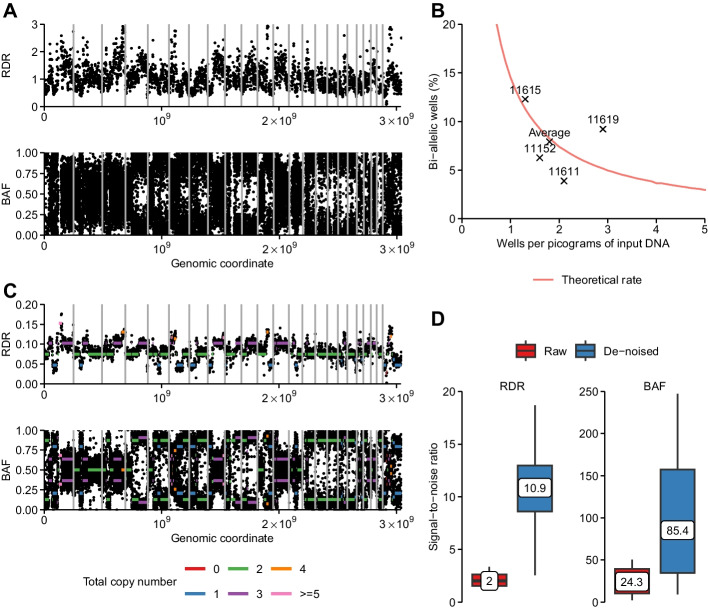
PicoCNV data de-noising. **A.** Raw RDR (top) and BAF (bottom) tracks for the DigiPico sample from patient 11152, derived from Illumina short read sequencing depths. RDR values were calculated in non-overlapping 1 Mb windows and corrected for GC content and mappability. BAF values were calculated at heterozygous SNPs. **B.** Percentage of bi-allelic wells predicted by theory (orange line) and observed for real samples (labelled crosses), as a function of the number of wells per input picograms of DNA. Theoretical predictions were obtained from a binomial distribution, assuming a random distribution of human genomes across multiple wells. Observed values were calculated as the percentage of wells containing both reference and alternative alleles at heterozygous SNPs. **C.** De-noised RDR and BAF tracks for the DigiPico sample from patient 11152. Coloured segments indicate copy number states fitted by PicoCNV. **D.** Per-patient signal-to-noise ratios (SNRs) for the RDR (left) and BAF (right). To calculate signal strength, data points in the copy number states {1,1} and {1,0} according to bulk ASCAT calls were compared. The signal was the squared difference in the means of these two sets. The noise was measured as the average within-segment variance, using segments taken from bulk ASCAT calls

The PicoCNV pipeline to call allele-specific CNAs from DigiPico data consists of two steps: 1. data de-noising and 2. CNA calling (Fig. 1). This two-stage approach was motivated by the observation that raw data from microscopic samples were unsuitable for copy number calling. To obtain CNAs from sequencing data, most algorithms use two quantities calculated along the genome: the read depth ratio (RDR, often transformed to the logR); and the B-allele frequency (BAF). When we calculated these quantities for microscopic samples from Illumina short reads, as is standard for bulk WGS data, they exhibited prohibitively high levels of noise (Fig. 2A). This confirmed the need for a preliminary data de-noising step in our pipeline.

To de-noise the RDR data, PicoCNV leverages DigiPico’s large mono-allelic fragments. DigiPico is a linked-read technology, in which large fragments of DNA (~ 100 kb) are distributed into wells, before being amplified, fragmented, barcoded and pooled for WGS [32] (Fig. 1). In practice, the Illumina short-read sequencing depth varies greatly between individual large DNA fragments due to non-uniform rates of amplification. This introduces a large amount of noise to the measurement of total sequencing depth along the genome. Since the RDR is calculated as the ratio of tumour and germline sequencing depths, this noise is reflected in the RDR. To counter this effect, PicoCNV reconstructs the original large DNA fragments in silico (Methods) and uses these to calculate the sequencing depth. The number of wells in DigiPico is sufficient for the input amount that large DNA fragments from overlapping loci are only rarely put into the same well (estimated < 10%, Fig. 2B). Therefore, each locus in the genome is covered by at most one large DNA fragment in the vast majority of wells. We call this the ‘mono-allelic’ property of DigiPico. Based on this, we reasoned that co-local Illumina short reads in the same well probably originated from the same large DNA fragment (Fig. 1). PicoCNV therefore uses the resulting in silico reconstructed large fragments (RLFs) to calculate the sequencing depth in DigiPico samples.

PicoCNV also de-noises the BAF data using DigiPico’s mono-allelic property. Phasing heterozygous single nucleotide polymorphisms (SNPs) into haplotypes separates the maternal and paternal alleles, allowing for aggregated calculation of allele frequencies [28, 33]. In DigiPico, SNPs that are near to each other and supported by overlapping sets of wells are likely to be on the same allele (Fig. 1). We used this observation to develop a phasing algorithm for DigiPico data (Methods). As with the RDR data, we observed that random variations in Illumina short read sequencing depth were a substantial source of noise. PicoCNV counteracts this noise by deriving BAF values from counts of wells rather than counts of short reads, in addition to haplotype aggregation (Methods).

Finally, PicoCNV calls CNAs using a well-established approach. It first segments the de-noised RDR and BAF data, before estimating the sample purity and ploidy, and finally fitting copy number states to each segment (Methods).

## Results

### DigiPico and matched bulk sequencing

To develop PicoCNV, we flow sorted small numbers of tumour-initiating cells (TICs) from four patients’ bulk tumour samples, and sequenced the TIC genomes with DigiPico (Methods, Additional file 1: Table S1). Each DigiPico run uses one or more 384-well plates, with more plates being used where more input DNA is available as appropriate to preserve DigiPico’s mono-allelic property (Fig. 2B). To probe PicoCNV’s robustness to reduced input DNA amounts, we used both single-plate and triplicate DigiPico runs, as well as analysing individual plates from the triplicate samples separately.

Flow sorting these microscopic tumour samples from bulk allowed us to simulate a scenario in which only tens or hundreds of cells were available, while keeping bulk data as a control against which to compare PicoCNV. However, we envisioned that PicoCNV would ultimately be used in settings where matched bulk samples are unavailable, such as MRD which we discuss later in this manuscript. To create a ground truth dataset for benchmarking, we performed WGS on the matched bulk samples. We then obtained bulk consensus CNA calls by combining the outputs of three established algorithms: ASCAT [14], Sequenza [15] and CNVkit [16] (Methods). By using multiple algorithms in this way, we aimed to have as little bias as possible in our ground truth data.

### De-noising with phase-based molecule counting

We applied PicoCNV’s de-noising steps to the TIC DigiPico data, which resulted in significantly cleaner RDR and BAF tracks along the genome compared to raw data (Figs. 2A, C). Quantitatively, we measured increases in the median per-sample signal-to-noise ratio of more than five-fold and thee-fold for the RDR and BAF, respectively (Fig. 2D, Additional file 1: Table S1). We were therefore satisfied that PicoCNV successfully leveraged the mono-allelic property of DigiPico to de-noise the RDR and BAF data.

### Purity and ploidy estimation

To produce accurate CNA calls, it is first crucial for any algorithm to correctly estimate the genome-wide average copy number (ploidy) and sample purity. A common difficulty is that high-ploidy solutions often fit data more closely than lower-ploidy ones, even if they do not reflect the true karyotype. To address this, we used a heuristic mean squared-error minimisation approach for purity and ploidy estimation, which selected approximately diploid solutions in all samples except one (Additional file 2: Figure S1, Methods). The remaining sample, from patient 11,611, was determined to be approximately tetraploid.

**Fig. 3:**
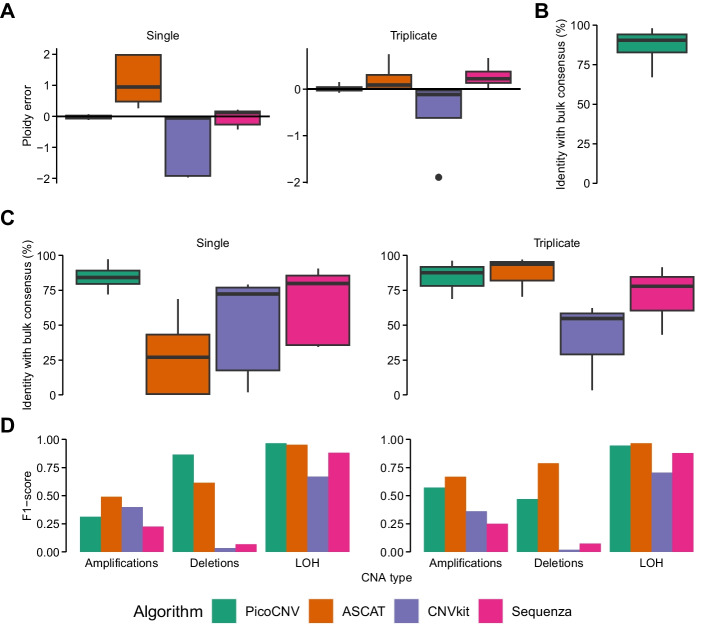
Accuracy benchmark of PicoCNV. **A** Absolute error in ploidy estimation relative to bulk consensus values, for single DigiPico plates (left) and triplicates (right). Distributions are across multiple samples. **B** PicoCNV accuracy on bulk WGS data, compared to bulk consensus CNA calls. **C** Percentage of the genome with copy number state exactly matching bulk consensus calls, for single DigiPico plates (left) and triplicates (right). **D** Overall F1-score to detect amplifications, deletions and LOH

We validated these ploidy estimates by comparing them to the bulk consensus data. PicoCNV was highly accurate, deviating by no more than 0.2 in any sample. By contrast, other algorithms that lacked PicoCNV’s specialised treatment of the DigiPico data gave substantially less accurate ploidies (Fig. 3A). In particular, ASCAT appeared to overestimate sample ploidy while CNVkit tended to underestimate it, although both of these tendencies were less pronounced for triplicate DigiPico samples. Indeed, ASCAT did not appear to be very robust on single-plate data, failing to solve the ploidy for two plates from sample 11,611 altogether. These results indicated that PicoCNV produced very accurate ploidy estimates that were uniquely robust to reduced amounts of input data.

### Copy number accuracy

While PicoCNV was designed for DigiPico sequencing data specifically, its copy number state estimation step could in theory be applied to any type of sequencing data. Therefore, we first tested it on our bulk WGS data to measure its baseline accuracy. We found that it had a 91% identity with the bulk consensus calls on average, confirming that it was capable of providing accurate copy number inferences given standard sequencing data (Fig. 3B).

Upon manual inspection of the de-noised DigiPico data, we found that a small portion of the genome contained apparently inconsistent RDR and BAF values (Additional file 2: Figures S2A, B). We reasoned that these loci may have undergone sub-clonal CNAs. While intra-sample heterogeneity was not the focus of PicoCNV, we allowed PicoCNV to call sub-clonal CNAs where fully clonal solutions were a poor fit, to prevent it being confused by such cases (Methods). To tune and assess PicoCNV’s sensitivity to sub-clonal CNAs, we ‘spiked in’ simulated events into real sample data (Methods). We found that CNAs present in around half of cancer cells were the most likely to be detected as sub-clonal, and we estimated that PicoCNV had 84.5% sensitivity to detect these CNAs (Additional file 2: Figure S2C). In practice however, more than 90% of the genome was determined to have fully clonal copy number states, as may be expected from flow-sorted samples comprising very few cells.

We then assessed the accuracy of PicoCNV’s copy number calls by comparing them to our ground truth data. We found that there was exact agreement on the copy number state between PicoCNV and bulk consensus data in 84% of the genome on average (Additional file 1: Table S1). On single DigiPico plates, PicoCNV significantly outperformed other algorithms, which likely suffered due to poor ploidy estimations (Fig. 3C). Interestingly, on triplicate DigiPico data PicoCNV’s performance was largely unchanged while ASCAT improved dramatically. This indicated that ASCAT was able to make accurate copy number inferences for sufficiently large DigiPico samples. However, PicoCNV demonstrated exceptional robustness to the reduced input single plate setting.

We further investigated PicoCNV’s ability to detect three categories of CNAs of general interest: amplifications, deletions, and loss of heterozygosity (LOH), in terms of the F_1_-score which combines sensitivity and specificity (Methods). On single DigiPico plates, PicoCNV performed better than alternative algorithms in detecting deletion and LOH events, while no algorithm had very high performance to detect amplifications (Fig. 3D). This could have reflected the fact that many amplifications were very short (e.g. focal amplifications), which are difficult to detect from picogram quantities of DNA. On triplicate data, PicoCNV did generally well (especially for amplifications and LOH events), but as with absolute copy number ASCAT improved dramatically with the additional input data. We concluded that PicoCNV was capable of detecting the majority of CNAs of interest, and that it was the most reliable algorithm when input data were limited to a single plate.

### Application to MRD

Having validated the PicoCNV pipeline, we applied it to a clinically relevant use-case where no bulk sample was available and only microscopic samples could be used. Minimal residual disease (MRD) in solid tumours refers to the deposits of cancer cells remaining after a patient has responded well to first-line treatments. MRD is a major source of disease recurrence, so being able to target these cells specifically has the potential to improve patients’ clinical outcomes. Indeed, so-called maintenance therapy has recently been adopted for some ovarian cancer patients [34]. We therefore sought to identify copy number biomarkers that might inform how a patient’s maintenance therapy would be managed.

**Fig. 4:**
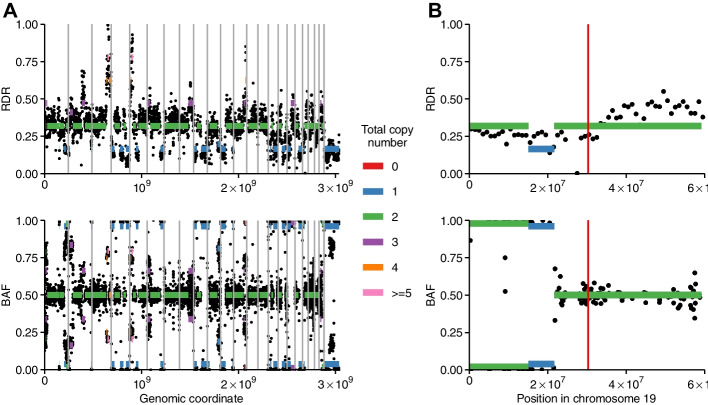
Application of PicoCNV to MRD. **A** De-noised RDR (top) and BAF (bottom) tracks for the MRD sample. Coloured segments indicate copy number states fitted by PicoCNV. Highlighted regions of the genome were determined to have undergone LOH. **B** RDR and BAF tracks for chromosome 19, with fitted PicoCNV copy number states. The vertical red line indicates the position of the *CCNE1* gene

We applied DigiPico sequencing and PicoCNV to an MRD sample taken from patient 11617 (Additional file 1: Table S1), obtained surgically as previously described [22]. PicoCNV produced visually clean RDR and BAF tracks along the genome (Fig. 4A), indicating that data de-noising was effective. Amplifications of *CCNE1* are known to indicate a poor prognosis in ovarian cancer [35]. Various therapies have been proposed specifically for *CCNE1*-amplified tumours, including WEE1 kinase and CDK2 inhibitors [35], and proteasome inhibitors [36]. However, in our MRD sample we found that *CCNE1* was not amplified, suggesting that such approaches would not be appropriate (Fig. 4B). A recent phase 3 clinical trial (ARIEL3) found that HRD is predicative of ovarian cancer response to PARP inhibitors for maintenance therapy [37]. The authors measured HRD using a combination of *BRCA* mutation status and the extent of genomic LOH. We found that patient 11617 was wild type for both *BRCA1* and *BRCA2*. We then used copy number calls from PicoCNV to measure the extent of LOH across the genome, and found that it was 31.1% (Fig. 4A). This would qualify as LOH-high in the ARIEL3 trial (threshold of 14%), suggesting that patient 11617 might respond well to PARP inhibitors for maintenance therapy. We concluded that PicoCNV was able to provide clinically useful insights in settings where only microscopic samples were available.

## Discussion

We have presented PicoCNV, a new copy number profiling protocol for microscopic cancer samples. PicoCNV uses the mono-allelic property of the DigiPico sequencing platform to de-noise the RDR and BAF data from microscopic tumour samples, before using this data to call CNAs. To our knowledge, this represents the first allele-specific CNA calling protocol that leverages linked-read technology, since previous methods have only provided total copy number [38]. We validated PicoCNV’s performance by showing that it had high agreement with ground truth results obtained from matched bulk tumour samples, and that it was significantly more robust than established algorithms in settings where input DNA amounts were the most reduced. Finally, we demonstrated the clinical utility of PicoCNV by showing how it could be used to inform maintenance therapy in the MRD setting.

This study faced two main limitations, in the nature of the patient cohort and the availability of ground truth data. The cohort of patients with available DigiPico sequencing data was small, consisting of only five patients. Combined with the fact that all samples were taken from ovarian cancers, this ran the risk of developing PicoCNV in a way that would generalise poorly to other data sets. However, our approach used assumptions that depended only on the sequencing platform itself. Moreover, ovarian cancer has one of the highest rates of genomic instability of any cancer type [11], and therefore may well have provided a particularly robust setting for developing a CNA calling pipeline. Nevertheless, more work with larger cohort sizes would further validate PicoCNV. Another challenge in this study was the lack of *bona fide* ground truth data. Using matched bulk data for this purpose was a reasonable choice, but it introduced two potential sources of error. First, while bulk WGS data is generally very reliable, calling CNAs from it is still challenging in some cases. Second, the cells used for DigiPico sequencing represented small numbers of cells taken from the matched bulk tumours by flow sorting. While it was reasonable to expect that copy number states were the same in the microscopic and bulk samples, it was possible that certain sub-clonal CNAs were disproportionately selected for or removed from the DigiPico input. Thus, we could not rule out the possibility of biological differences between the matched DigiPico and bulk samples. The accuracy of PicoCNV that we measured therefore likely represents a lower bound on its true performance.

Calling CNAs accurately from microscopic tumour samples is challenging. Single cell methods can provide an alternative approach, depending on researchers’ requirements. For example, they provide unparalleled insights into intra-tumour heterogeneity. We note two main differences in the applicability of DigiPico and single cell sequencing approaches. First, single cell methods often need to aggregate data across thousands of individual cells. This is particularly true for shallow sequencing (< 0.1 × per cell), in which individual cells simply do not have sufficient data to call CNAs at high resolution. On the other hand, we have demonstrated here that DigiPico and PicoCNV can obtain accurate CNA calls starting from as few as 40 cells (Additional file 1: Table S1, assuming 6 pg of DNA per cell). Second, a complete genomic characterisation of cancer comprises both copy number states and small somatic mutations (SSMs), *i.e.* SNVs and indels. Obtaining accurate SSMs from single cell data remains challenging. By contrast, we have developed a machine learning approach, MutLX, to identify somatic SSMs from DigiPico sequencing data [32]. Internal benchmarking currently indicates that MutLX has a sensitivity of 76% and specificity of 95% for detecting SSMs (Additional file 1: Table S1), and this will be the subject of a future manuscript. Combined then, PicoCNV and MutLX will allow for a complete genomic characterisation of cancer starting from microscopic tumour samples. To our knowledge, this is not currently feasible with single cell approaches. Finally, we note that the accuracy of single cell CNA calling methods has generally not been assessed with direct comparison to bulk data, likely due to their emphasis on intra-tumour heterogeneity. Instead, they have been assessed using heuristics [27, 28], simulation [26] and sub-sampling [29].

## Conclusions

We have demonstrated that PicoCNV is able to accurately and robustly call allele-specific somatic CNAs from microscopic tumour samples. Future studies could use PicoCNV to study minimal residual disease (MRD) and circulating tumour cells. They could also explore potential applications in liquid biopsies. We could additionally use our platform to examine how acquired resistance to therapies evolves at the MRD stage. In ovarian cancer in particular, where recurrence rates after standard of care are very high (70–80%) [39], this remains a pressing question.

## Methods

### Patient samples

Written consent from study participants was obtained for participation in the prospective biomarker validation study Gynaecological Oncology Targeted Therapy Study 01 (GO-Target-01) under research ethics approval number 11/SC/0014. Tumour and blood samples were obtained on the day of surgery. Blood samples were processed to isolate peripheral blood mononuclear cells (PBMCs) using Lymphoprep™ (STEMCELL Technologies, Canada). Tumour tissue was dissociated using human Tumor Dissociation Kits (Miltenyi Biotec, Germany) following the manufacturer’s protocol. Dissociated tumour cells were processed for staining and fluorescent activated cell sorting to obtain bulk cancer cells and tumour initiating cells (TICs) as described in Okamoto et al*.* [40].

### DNA extraction and WGS library preparation

DNA from PBMCs and bulk cancer cells was isolated using DNeasy blood and tissue kit (Qiagen, USA). Illumina whole-genome sequencing libraries for both germline and tumour DNA were constructed and sequenced by Novogene Co. Ltd (China).

### DigiPico library prep and sequencing

For DigiPico sequencing, DNA was isolated from TICs using Repli-g single cell kits (Qiagen, USA). DigiPico library prep was performed as described in Carrami et al*.* [32]. The libraries were then sent to a sequencing company (Novogene Co. Ltd, China) and were sequenced on a Novaseq platform in 150 bp paired-end sequencing.

### Sequencing data pre-processing

Bulk tumour and normal blood-derived whole genome sequencing data were trimmed of Illumina adapters with Trim Galore v0.6.6 [41] and aligned to the human genome version hg19 using Bwa-mem v2.2.1 [42]. PCR duplicate reads were removed from the resulting BAM files using Picard Tools v2.18.17 [43].

For DigiPico data, raw paired-end whole genome sequencing data were demultiplexed to give two FASTQ files per well using custom scripts. Reads from each well were then trimmed of Illumina adapters using Trim Galore v0.6.6 [41] and aligned to the human genome version hg19 using Bowtie v2.4.1 [44]. The resulting BAM files were cleaned of PCR duplicates with Picard Tools v2.18.17 [43]. The per-well BAM files were merged with samtools, using read groups to keep track of the wells that each read originated from.

### ASCAT implementation

A set of common SNPs was obtained from HapMap v3.3 [45]. These were genotyped in bulk tumour and matched germline WGS data using Platypus [46] to produce allele-specific read counts in both samples. Retained heterozygous SNPs consisted of those with at least 20 covering reads and an allele frequency of between 25 and 75% in the germline sample. Tumour and germline BAFs were calculated for these SNPs from the read counts. LogR was calculated at each SNP as $\log_{2} \left( {\frac{{{\text{tumour}}\;{\text{reads}}}}{{{\text{germline}}\;{\text{reads}}}}} \right)$, and centred to have zero median for each patient.

ASCAT [14] v2.5.2 was obtained from Bioconda [47]. The ‘Standard ASCAT run’ pipeline from https://github.com/VanLoo-lab/ascat/tree/master/ExampleData was used to call CNAs, including GC-wave correction and with the compaction parameter gamma set to 1.

### CNVkit implementation

CNVkit [16] v0.9.10 was obtained from Bioconda [47]. For each sample, a VCF of heterozygous SNPs and their allele-specific read depths was obtained. The CNVkit ‘batch’ pipeline described at https://cnvkit.readthedocs.io/en/stable/pipeline.html was followed in WGS mode, with the VCF provided to the CNVkit call command for allele-specific copy number calls.

### Sequenza implementation

Sequenza [15] v3.0.0 was obtained from Bioconda [47]. The ‘Normal and tumor BAM files’ pre-processing described at https://sequenza-utils.readthedocs.io/en/latest/guide.html was followed, binning data to 1 Mb. Copy number segments were then fit in R as described at https://bitbucket.org/sequenzatools/sequenza.

### Bulk consensus copy number calls

CNA calls from ASCAT, CNVkit and Sequenza on bulk WGS were combined into a consensus call set for each sample. First, ploidies were matched by popular vote. For example, if two algorithms found diploid solutions and one found a tetraploid solution, the latter algorithm was re-run with a forced diploid solution. Copy number states were then obtained by taking the median of the major and minor copy numbers called by the three algorithms at each point in the genome.

### PicoCNV RDR calculation

The original large DNA fragments from the DigiPico protocol were reconstructed in silico to give reconstructed large fragments (RLFs). Separately for each chromosome in each well, read pairs were determined to come from the same RLF if the gap between them was less than 100 kb. Each RLF spanned the from start of the first read belonging to it, to the end of the last read.

The PicoCNV read depth ratio (RDR) was calculated in non-overlapping 1 Mb windows along the genome for DigiPico samples. Within each window, the raw RDR was calculated as $r = 100 \times \frac{{{\text{average}}\;{\text{RLF}}\;{\text{depth}}}}{{{\text{germline}}\;{\text{reads}}}}$. It was then corrected for GC content and mappability using a multivariate linear model trained separately for each sample.

### PicoCNV SNP phasing and BAF calculation

A set of common SNPs was obtained from dbSNP v138 [48], and retained as heterozygous for each patient if in the germline data they had a sequencing depth of at least 20 reads and an allele frequency of between 25 and 75%. Phasing each patient’s heterozygous SNPs from DigiPico tumour sequencing data consisted of two steps: partitioning the genome into blocks; and assigning SNPs within each block to one of two haplotypes.

Potential blocks of SNPs were first identified as contiguous regions where the gap from one SNP to the next was no greater than 500 kb. Blocks containing more than 100 SNPs were subdivided so that they did not exceed this limit. Blocks were then checked for connectivity. A block was disconnected if there existed a sub-partitioning such that the SNPs in each partition did not share any covering wells with the other partitions. Disconnected blocks were sub-divided into these partitions. Finally, any remaining singleton blocks consisting of only one SNP were removed.

Within each block, SNPs were then assied to one of two haplotypes. To do this, the SNP-SNP similarity matrix $M$ was first constructed as

$$M_{ij} = \frac{{\mathop \sum \nolimits_{k} G_{ik} G_{jk} }}{{\mathop \sum \nolimits_{l} \left| {G_{il} } \right|\left| {G_{jl} } \right| }}, \;{\text{where}}\; G_{ik} = \left\{ {\begin{array}{*{20}c} { + 1,} & {if\;SNP\; i\; is\; alt\; in\; well\; k} \\ { - 1,} & {if\; SNP\; i\; is\; ref\; in\; well\; k} \\ {0,} & {if\; SNP\; i\; is\; not\; covered\; by\; well\; k} \\ \end{array} } \right..$$

$M$ contains values between $+1$ and $-1$, with ${M}_{ij}=+1$ if SNPs $i$ and $j$ have the same status in all wells, and ${M}_{ij}=-1$ if they are different in all wells. It can be shown that, if the mono-allelic property of DigiPico holds exactly, then ${M}_{ij}={h}_{i}{h}_{j}$, where $h$ is a haplotype vector indicating which haplotype each SNP belongs to with entries $\pm 1$. By convention, we always take the first entry in $h$ to be positive. This matrix outer product can be efficiently computed by applying singular value decomposition to $M$. In particular, the first singular vector ${h}^{*}$ of $M$ is a close approximation to $h$, and the final estimate of $h$ is ${h}_{i}=\mathrm{sgn}\left({h}_{i}^{*}\right)$.

The PicoCNV BAF value was then calculated for each haplotype group as

$$b = \frac{{\mathop \sum \nolimits_{{{\text{SNPs}}}} \;{\text{wells}}\;{\text{supporting}}\;{\text{alt}}\;{\text{allele}}}}{{\mathop \sum \nolimits_{{{\text{SNPs}}}} \;{\text{wells}}\;{\text{covering}}\;{\text{SNP}}}}$$

using the median genomic coordinate of all SNPs in the group as the position of the group.

### Segmentation

The genome was segmented first using the BAF data alone, and then further segmented using the RDR data alone. This reflected the observation that the BAF data tended to be cleaner than the RDR data, even after data de-noising. Prior to segmentation, BAF data $b$ were mirrored according to ${b}_{\mathrm{mirr}}=0.5-\left|0.5-b\right|$, and RDR data $r$ were normalised according to $r_{{{\text{norm}}}} = r/4\overline{r}$. Here, $\overline{r}$ is the genome-wide average of the corrected RDR. This normalisation ensured that the RDR and mirrored BAF data were on comparable scales.

Segmentation was performed in each chromosome arm separately. Segmentation of data ${\varvec{x}}$ in each arm was performed by minimising the loss function

$$L\left({\varvec{x}}\right)=\sum_{s\in S}\sum_{i\in s}{\left({w}_{i}{x}_{i}-{\overline{x} }_{s}\right)}^{2}+\lambda \left|S\right|$$

Here, $s$ indexes segments, $S$ is the set of all segments on the chromosome arm, ${w}_{i}$ is a normalised weight per data point (size of haplotype group for BAF data, 1 for RDR data), ${\overline{x} }_{s}$ is the per-segment weighted mean, and $\lambda$ is a parameter that we set to 0.1. Segment lengths were kept above a minimum of 5 Mb. Minimisation was carried out by a stochastic greedy search algorithm. To ensure that we found a global minimum of the loss function, we implemented the greedy search 10,000 times and took the best resulting segmentation.

### Purity and ploidy grid search

For a range of values of sample purity $\phi$ and tumour ploidy $\psi$, the genome-wide mean squared error (MSE) was calculated. This MSE was calculated as a length-normalised sum over segments,

$${\text{MSE}} = \frac{{\mathop \sum \nolimits_{s} length\left( s \right) {\text{MSE}}_{s} }}{{\mathop \sum \nolimits_{s} length\left( s \right)}}$$

Within each segment $s$, the MSE was a sum of terms from the RDR and mirrored BAF,

$${\text{MSE}}_{s} = {\text{MSE}}_{s}^{\left( r \right)} + {\text{MSE}}_{s}^{\left( b \right)}$$

with

$${\text{MSE}}_{s}^{\left( x \right)} = \frac{1}{\left| s \right|}\mathop \sum \limits_{i \in s} \frac{{\left( {x_{i} - \hat{x}_{s} } \right)^{2} }}{{{\widehat{\sigma }}_{x}^{2} }}$$

Here, $\left|s\right|$ is the number of data points in $s$, ${\widehat{\sigma }}_{x}^{2}$ is a genome-wide estimate of the variance of $x$ using deviation from per-segment means, and the estimators ${\widehat{x}}_{s}$ for RDR and BAF are functions of the copy number state. If a state consists of major and minor copy numbers ${n}_{A}$ and ${n}_{B}$ respectively, then

$$\widehat{r}_{s} = \overline{r}\frac{{\phi \left( {n_{A} + n_{B} } \right) + 2\left( {1 - \phi } \right)}}{{\phi \psi + 2\left( {1 - \phi } \right)}} \;{\text{and}}\; \widehat{b}_{s} = \frac{{\phi n_{B} + \left( {1 - \phi } \right)}}{{\phi \left( {n_{A} + n_{B} } \right) + 2\left( {1 - \phi } \right)}}$$

assuming that non-cancer cells are diploid. A finite set of copy number states were considered for each segment. This set consisted of all possible states with total copy number up to the largest value consistent with the RDR observed for the entire genome. In each segment, the state with the smallest MSE was selected, and the resulting whole-genome MSE was then calculated as detailed above.

Selecting the $\left(\phi ,\psi \right)$ pair with the minimal MSE would often result in erroneously tetraploid solutions. To counter this, we identified local MSE minima from the grid search. If it was sufficiently pronounced, the lowest-ploidy MSE minimum was taken as the solution (Additional file 2: Figure S1B).

### Sub-clonal copy number fitting

Given values for $\phi$ and $\psi$ from the grid search, final copy number states were fit using a modified version of the MSE-minimisation approach above. In particular, the set of possible copy number states for each segment was expanded to include clonalities $\chi \in \left\{0.5, 0.6, 0.7, 0.8, 0.9, 1\right\}$. We modelled tumour cells without a particular CNA as having the modal copy number state $\left\{{m}_{A},{m}_{B}\right\}$ from the fully clonal solution. The RDR and BAF estimators were therefore changed to

$$\begin{aligned} \widehat{r}_{s} & = \overline{r}\frac{{\phi \chi \left( {n_{A} + n_{B} } \right) + \phi \left( {1 - \chi } \right)\left( {m_{A} + m_{B} } \right) + 2\left( {1 - \phi } \right)}}{{\phi \psi + 2\left( {1 - \phi } \right)}} \\ \widehat{b}_{s} & = \frac{{\phi \chi n_{B} + \phi \left( {1 - \chi } \right)m_{B} + \left( {1 - \phi } \right)}}{{\phi \chi \left( {n_{A} + n_{B} } \right) + \phi \left( {1 - \chi } \right)\left( {m_{A} + m_{B} } \right) + 2\left( {1 - \phi } \right)}} \\ \end{aligned}$$

To avoid over-fitting of sub-clonal solutions, a penalty term was added to the per-segment MSE so that

$${\text{MSE}}_{s} = {\text{MSE}}_{s}^{\left( r \right)} + {\text{MSE}}_{s}^{\left( b \right)} + \pi \left( \chi \right),\;{\text{where}}\; \pi \left( \chi \right) = \left\{ {\begin{array}{*{20}c} {0,} & {if\;\chi = 1} \\ {1,} & {otherwise} \\ \end{array} } \right.$$

The copy number state with the smallest value of ${\mathrm{MSE}}_{s}$ was chosen for each segment.

### Sub-clonal simulation and sensitivity calculation

To assess PicoCNV’s sensitivity to detect sub-clonal CNAs and tune its sub-clonal penalty term $\pi$, CNAs were artificially ‘spiked in’ to real data. First, the mean and variance of each copy number state in each sample was determined from fully-clonal PicoCNV solutions. Segments were then repeatedly chosen at random and overwritten with simulated data reflecting a random subclonal copy number state. For a CNA with clonality $\chi$, the RDR data $r$ were simulated as

$$r \sim \chi N\left({\overline{r} }_{CNA},{\sigma }_{CNA}^{2}\right)+\left(1-\chi \right)N\left({\overline{r} }_{modal},{\sigma }_{modal}^{2}\right)$$

where $N$ denotes the normal distribution, ${\overline{r} }_{CNA}$ and ${\sigma }_{CNA}^{2}$ are the mean and variance of the CNA being simulated, and ${\overline{r} }_{modal}$ and ${\sigma }_{modal}^{2}$ are the mean and variance of the background modal copy number state for the sample. For example, a diploid sample would typically have background copy number state {1,1}.

The sensitivity was calculated as the percentage of simulations where PicoCNV correctly detected the simulated CNA and determined that it was sub-clonal.

### Performance for amplifications, deletions and LOH

For each sample, the median total copy number ${n}_{\mathrm{med}}$ was calculated. Amplifications were then identified as contiguous regions with total copy number $\ge 2{n}_{\mathrm{med}}$. Deletions were regions with total copy number 0, and LOH was defined as ${n}_{B}=0$ with ${n}_{A}>0$. We measured an algorithm’s sensitivity to detect these events from DigiPico data as

$$\frac{{\# { }\;{\text{bases}}\;{\text{where}}\;{\text{algorithm}}\;{\text{and}}\;{\text{bulk}}\;{\text{consensus}}\;{\text{agreed }}\;{\text{on }}\;{\text{event}}}}{{\# \;{\text{bases}}\;{\text{where }}\;{\text{bulk}}\;{\text{ consensus }}\;{\text{called}}\;{\text{ the}}\;{\text{event}}}},$$

and its specificity as

$$\frac{{\# { }\;{\text{bases }}\;{\text{where }}\;{\text{algorithm }}\;{\text{and}}\;{\text{bulk}}\;{\text{consensus}}\;{\text{agreed}}\;{\text{on}}\;{\text{event}}}}{{\# \;{\text{bases }}\;{\text{here}}\;{\text{algorithm }}\;{\text{called }}\;{\text{the}}\;{\text{event}}}}.$$

From these, we calculated the F1-score as

$$F_{1} = \frac{{2 \times {\text{sensitivity}} \times {\text{specificity}}}}{{{\text{sensitivity}} + {\text{specificty}}}}.$$

### Availability and requirements

Project name: PicoCNV. Home page: https://process.innovation.ox.ac.uk/software. Operating system: Linux. Programming languages: Python, R, Shell. Other requirements: Snakemake v6 or higher, Conda. License: Academic-use. Any restrictions to use by non-academics: License needed.

### Supplementary Information

**Additional file 1**. **Table S1**: Study cohort and per-patient results

**Additional file 2**. Supplementary figures S1 and S2.

## Data Availability

Binary alignment map (BAM) files containing the germline, bulk tumour and DigiPico sequencing data analysed in this manuscript are stored on the European Genome-Phenome Archive with accession numbers EGAD00001005118 (patient 11,152) and EGAD00001010302 (patients 11,611, 11,615, 11,619 and 11,617).

## Abbreviations

BAF: B-allele frequency
CNA: Copy number alteration
MRD: Minimal residual disease
RDR: Read depth ratio
RLF: Reconstructed large fragment
SNP: Single nucleotide polymorphism
SSM: Small somatic mutation
WGS: Whole genome sequencing
