# Correction: Completing a genomic characterisation of microscopic tumour samples with copy number

**DOI:** 10.1186/s12859-024-05642-8

**Published:** 2024-01-12

**Authors:** Joel Nulsen, Nosheen Hussain, Aws Al-Deka, Jason Yap, Khalil Uddin, Christopher Yau, Ahmed Ashour Ahmed

**Affiliations:** 1 Weatherall Institute for Molecular Medicine, University of Oxford, Oxford, UK; 2 Nuffield Department for Women’s and Reproductive Health, University of Oxford, Oxford, UK; 3 Singula Bio Ltd., Oxford, UK; 4 University of Birmingham, Birmingham, UK; 5 Health Data Research UK, London, UK; 6 Oxford Biomedical Research Centre, National Institute of Health Research, Oxford, UK

**Correction: BMC Bioinformatics (2023) 24:453** 10.1186/s12859-023-05576-7

Following the publication of the original article [1], the authors identified errors in affiliation 5 and funding statement. The correct affiliation and funding are given below.

The incorrect affiliation is: 5 Health Data Research UK, Oxford, UK

The correct affiliation is: 5 Health Data Research UK, London, UK

The incorrect funding is: This work was funded by Ovarian Cancer Action grant numbers HER00760 and HER01150, as well as by Singula Bio Ltd

The correct funding is: This work was funded by Ovarian Cancer Action grant numbers HER00760 and HER01150, as well as by Singula Bio Ltd. CY is supported by an EPSRC Turing AI Acceleration Fellowship (Grant Ref: EP/V023233/2).

The original article [1] has been corrected.
