# Supplementary material for: Completing a genomic characterisation of microscopic tumour samples with copy number
Source: BMC Bioinformatics. 2023 Nov 30;24:453. doi: 10.1186/s12859-023-05576-7 (PMC10688092; doi:10.1186/s12859-023-05576-7)
Supplement: Supplementary file 2 — Additional file 2. Supplementary figures S1 and S2. [file 12859_2023_5576_MOESM2_ESM.pdf]

PicoCNV Supplementary Figures

Figure S1: Purity/ploidy grid search 2

Figure S2: Sub-clonal CNAs in DigiPico data 3

Figure S1: Purity/ploidy grid search

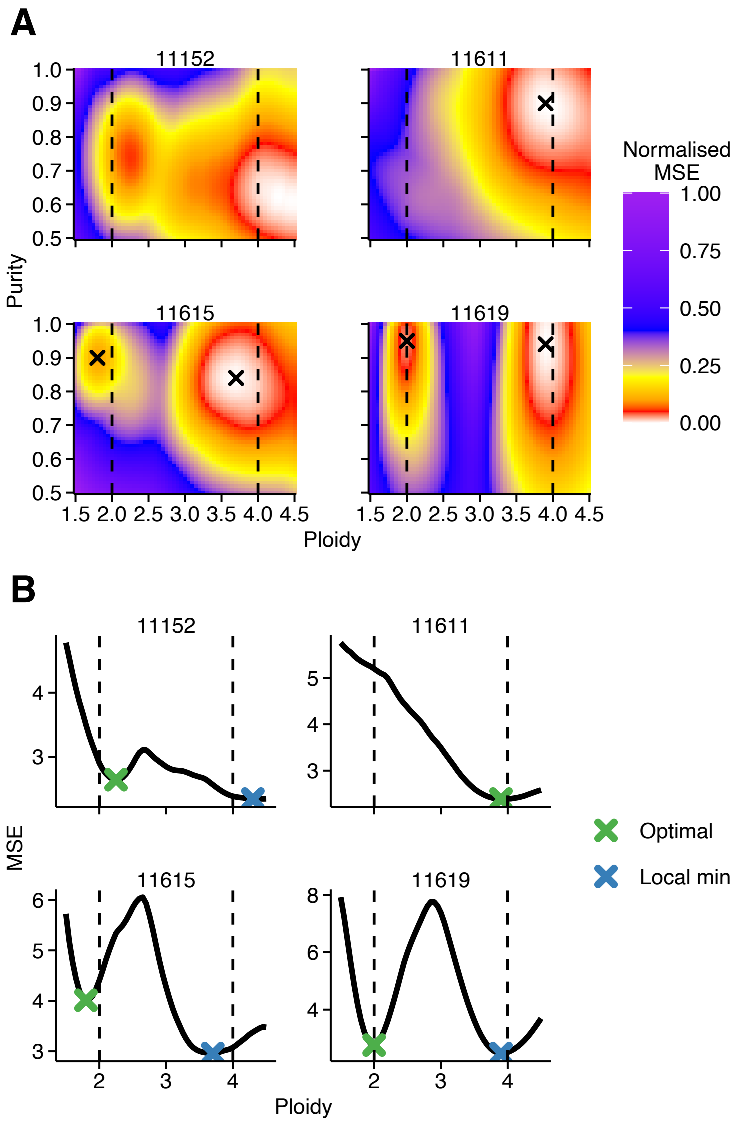

A. Whole-genome MSE values for each purity/ploidy value pair. Within each patient, the range of MSE values was min-max normalised for display purposes. Vertical dotted lines indicate the location of diploid and tetraploid solutions.

B. Illustration of ploidy selection. For each ploidy value, the optimal purity according to MSE was selected to create a 1-dimensional selection problem. Local minima on the resulting curve were then identified. For curves with two minima, their heights relative to the interposing maximum were calculated. If the height of the first minimum was at least one half the height of the second, then the first minimum was selected as the sample ploidy.

Figure S2: Sub-clonal CNAs in DigiPico data

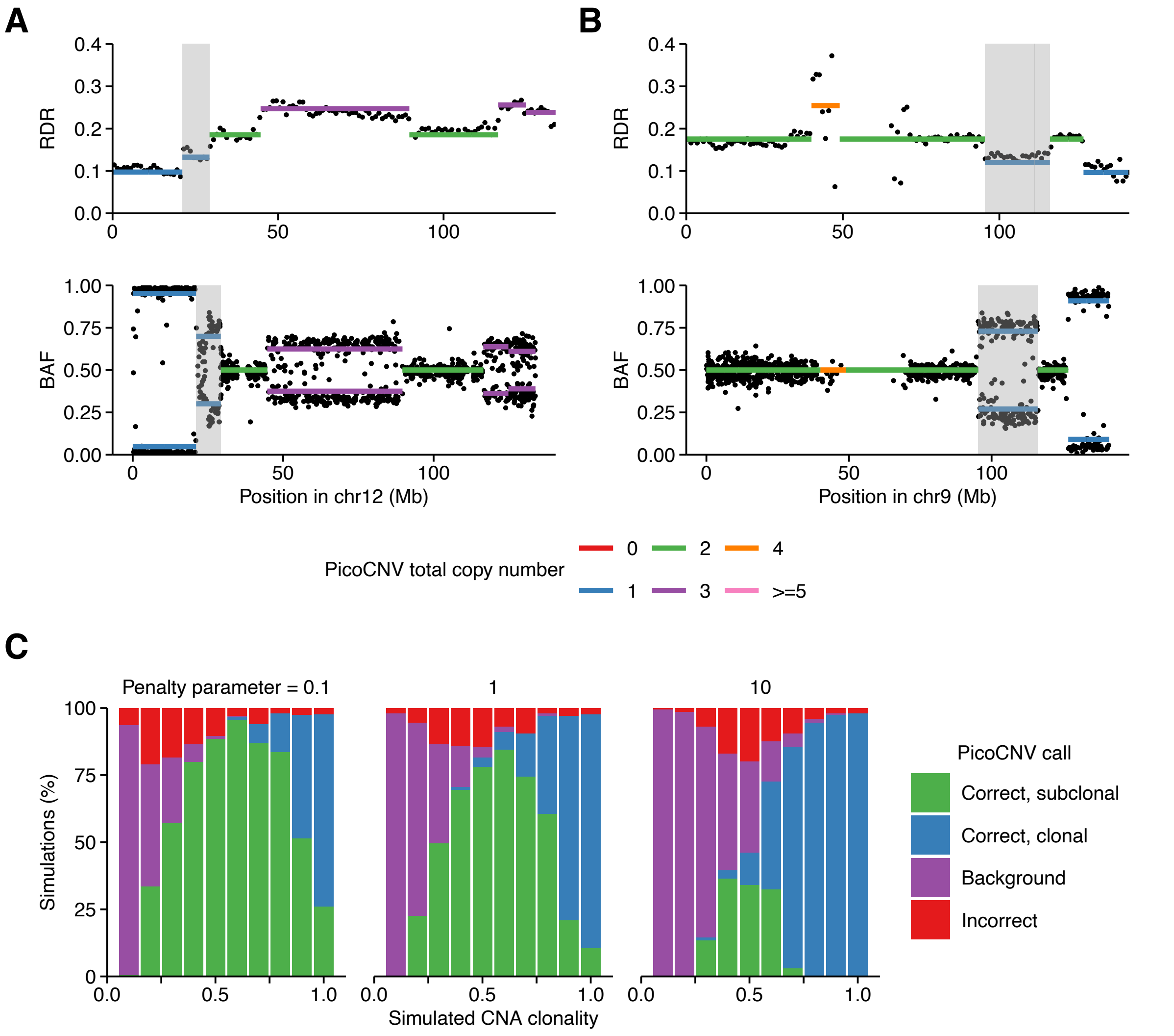

Examples of inconsistent RDR and BAF values from patients 11619 (**A**) and 11615 (**B**). Data for entire chromosomes are shown with the exemplar regions shaded. In both cases, the RDR is between the typical levels for copy number 1 and 2, while the BAF values indicate an unbalanced mix of alleles. These therefore represent sub-clonal heterozygous deletions.

C. Simulation results for sub-clonal CNA sensitivity estimation. Sub-clonal calls were penalised by a model parameter, increasing from 0.1 in the left panel to 10 in the right panel. Calls were determined to be incorrect if they reflected neither the CNA being simulated nor the background copy number for the sample. The parameter value of 1 (middle panel) give the lowest overall proportion of incorrect CNA calls (7.35%) while

retaining good sensitivity for sub-clonal CNAs, peaking at 84.5% for CNAs with clonality 0.6. It was therefore chosen as PicoCNV's default parameter value.
